# Interoceptive grounding of conceptual knowledge: new insight from an interoceptive-exteroceptive categorization task of concepts

**DOI:** 10.1007/s00426-025-02155-8

**Published:** 2026-01-03

**Authors:** Laura Barca, Salvatore M. Diana, Daniela Coutiño Duarte, Giuseppina Porciello, Anna M. Borghi

**Affiliations:** 1 Institute of Cognitive Sciences and Technologies, National Research Council, ISTC-CNR, Via G. Romagnosi 18A, 00196 Rome, Italy; 2 Universität Zu Köln, Colonia, Germany; 3 IRCCS Fondazione Santa Lucia Research Hospital, Rome, Italy; 4 Department of Psychology, Sapienza University of Rome, Rome, Italy; 5 Department of Dynamic and Clinical Psychology, and Health Studies, Sapienza University of Rome, Via Dei Marsi 78, 00185 Rome, Italy

## Abstract

The role of interoception, the sensing of internal bodily signals, in shaping our understanding of concepts remains an intriguing and understudied area of research. Here, we investigate the interoceptive foundation of conceptual representation, particularly for abstract concepts compared to concrete ones. Using a novel mouse-tracking paradigm, participants categorized various types of abstract and concrete concepts (i.e., abstract emotional, abstract philosophical, concrete natural and concrete artifact) as interoceptive (i.e., experienced through internal bodily sensations) or exteroceptive (i.e., experienced through the five perceptual senses). Results on the reaction times show that abstract-emotional concepts were more readily classified as interoceptive than abstract-philosophical concepts, emphasizing the importance of the interoceptive dimension for this category. Movement trajectories showed the implicit activation of interoceptive features also during the categorization of concrete natural concepts. To account for individual differences in interoceptive accuracy (i.e., the ability to accurately perceive visceral signals), participants performed a cardiac interoceptive task (i.e., the heartbeat counting task). Higher interoceptive accuracy was associated with faster categorization speeds, particularly for concrete-natural concepts. Taken together, our findings emphasize the multifaceted nature of conceptual knowledge where the interoceptive dimension plays a key role.

**Supplementary Information:**

The online version contains supplementary material available at 10.1007/s00426-025-02155-8.

## Introduction

Conceptual knowledge forms the foundation of how we understand the world and our experiences. Concepts, i.e., the basic unit of knowledge, help people distinguish and recognize objects and entities, infer their properties, and prepare to interact with them. Traditionally, scholars have distinguished between concrete concepts, having a perceptually bounded, single referent (e.g., table), and abstract ones (e.g., freedom), (for this distinction, see Banks et al., 2023; Borghi & Fernyhough, 2023; Reilly et al., 2023). In line with embodied cognition theories, showing that even seemingly abstract processes (such as grammar) can be biased by the signals coming from the body, we explore the following questions: What role do our body's signals play in shaping concepts? Specifically, do interoceptive signals (from the viscera) and exteroceptive signals (from the five senses) contribute differently to conceptual knowledge? If so, how might these differing contributions affect the processing of different concepts, as concrete and abstract, and their respective subcategories?

Traditionally, exteroception (i.e., our five senses: sight, touch, taste, smell, and hearing) has dominated the conversation about grounding concepts – how sensory experiences give meaning to abstract ideas. Studies with various methods (e.g., free listing, ratings, semantic priming, lexical decision, and feature verification tasks) have shown that concepts, particularly concrete ones, activate perception and action and often elicit multiple sensory modalities simultaneously (Borghi & Barsalou, 2021; Fernandino et al., 2016; Lynott et al., 2020; Petilli et al., 2021; Villani et al., 2021). Importantly, recent evidence suggests that sensorimotor aspects characterize not only concrete concepts but also abstract ones (Banks & Connell, 2023).

However, recent research suggests that interoception - the sensing of the internal state of our body - goes beyond the regulation of physiological states (Barrett & Simmons, 2015; Tsakiris & Critchley, 2016) and it is increasingly recognized as central to the experiences of agency and selfhood (Seth & Tsakiris, 2018), emotional and affective states (Barrett, 2017; Porciello et al., 2024; Seth, 2013) and mental health and wellbeing (Barca, 2025; Barca et al., 2023b; Maisto et al., 2021; Singh Solorzano et al., 2022; Vabba et al., 2023). The choice of interoceptive measures in our study is based on several key considerations. Notably, interoception is not a unidimensional construct but rather comprises distinct, independent dimensions, each requiring specific assessment (Garfinkel et al., 2015). To capture this complexity, we adopted the multidimensional model proposed by Garfinkel et al. (2015), which conceptualizes interoception through: Interoceptive accuracy – measured via behavioral tasks; Interoceptive sensibility – assessed through self-reports; Interoceptive awareness – evaluated by comparing behavioral and self-reported measures. Here, we choose to rely on the accuracy and sensibility measures of interoception. Thus, interoception, similar to exteroception, might play a prominent role in the conceptual representations of concepts, particularly emotional and abstract ones (Diveica et al., 2024). For example, Connell and colleagues (Connell et al., 2018) analyzed ratings on the five senses and interoceptive dimensions on more than 32.000 English concrete and abstract words. They found that the interoceptive dimension is a critical modality in the experience of abstract concepts. Villani (2021) confirmed the importance of the interoceptive dimension for abstract concepts via a dual task in which participants had to pay attention to inner bodily signals by monitoring their heart-beating pace and concurrently rate the difficulty of concrete and abstract concepts. In this task, the perceived difficulty of abstract concepts increased compared with that of concrete concepts.

Among abstract concepts, emotional concepts represent a particularly interesting category for the study of interoception. They lack the sensorimotor grounding of concrete concepts but have an important physiological component, which prevents them from being detached from sensory experiences (Mazzuca et al., 2017). Therefore, the relationship between interoception and emotional concepts could be more straightforward. In line with this idea, recent findings suggest that our physiological state (Pezzulo et al., 2018; Yu et al., 2021), as well as individual differences in conceptual knowledge of emotions (Brooks & Freeman, 2018), modulate the perception and categorization of facial emotion expression.

To the best of our knowledge, at the moment, no one has tested the implicit role of the interoceptive dimension in conceptual representation; thus, one of the goals of the present work is to explore the interplay between inner bodily sensations and higher-order cognitive processes by 1) developing a new behavioral paradigm of ‘interoceptive-exteroceptive’ categorization of various types of concrete and abstract concepts; and 2) testing whether participants’ performance at this new task is modulated by their interoceptive abilities.

To ensure clarity, we differentiate between ‘interoception,’ the process of sensing internal bodily states, and ‘interoceptive,’ an adjective describing aspects related to this process. For instance, in our task, participants categorized concepts as ‘interoceptive’ if they perceived them as grounded in internal bodily sensations, thus reflecting the *interoceptive* nature of those concepts*.*

Participants were presented with different kinds of (abstract and concrete) concepts and were asked to indicate - by moving the computer mouse - whether they perceived them by inner bodily sensations (i.e., interoceptive) or by the five perceptual senses (i.e., exteroceptive).

The categorization paradigm has been developed within the Mouse Tracker software (Freeman & Ambady, 2010), allowing the gathering of kinematic measures of the decision, in the temporal domain (overall response time, movement initiation time), in the spatial domain (trajectory area under the curve and maximum deviation, indexing the degree of competition exerted by the alternative response). Mouse tracking has been extensively utilized in psychology research, effectively elucidating the dynamic processes of conflict within the psycholinguistics domain (Barca & Pezzulo, 2012, 2015; D’Aversa et al., 2022), embodied (Flumini et al., 2015), social cognition (Freeman & Ambady, 2009; Smeding et al., 2016), visual perception (Quétard et al., 2016; Quinton et al., 2014), decision-making (Iodice et al., 2017) and affective-emotional processes (Barca et al., 2023a, b; Pezzulo et al., 2018; Yu et al., 2021). It offers several advantages over traditional aggregate response time measures, such as providing millisecond-resolution timing information (see Freeman, 2018) for an in-depth discussion). Analyzing response trajectories and related indices enables the examination of competitive mechanisms between response alternatives or attraction toward a temporally considered response that has not been explicitly selected, hence the activation of interoceptive/exteroceptive features (Barca & Pezzulo, 2012, 2015; Connell et al., 2018).

On the basis of previous evidence on the differential recruitment of interoceptive features in conceptual processing (Connell et al., 2018; Diveica et al., 2024; Falcinelli et al., 2024; Villani et al., 2021), we selected different kinds of abstract concepts, namely abstract-philosophical (e.g., ‘destiny’) and abstract-emotional (e.g., ‘infancy’) concepts, with the latter presumably eliciting interoceptive information to a greater extent. Concrete concepts were artifacts (e.g., ‘shop’) and natural kinds (e.g., ‘cave’), both of which were experienced primarily through perception. We selected artifacts and natural ones since they are the most commonly used in studies on conceptualization (Forde & Humphreys, 2005; Warrington & Shallice, 1984). Notably, we selected natural concepts without animacy cues, to avoid confounding. Thus, going to the aforementioned greater reliance of abstract concepts on interoceptive processes (Connell et al., 2018), we established that, within our two-choice decision paradigm, abstract concepts belong primarily to the ‘interoceptive’ category and should be categorized as such (i.e., perceived more through internal bodily sensations), whereas concrete concepts should be categorized as ‘exteroceptive’ (i.e., perceived through sensory perception). In this context, cases where abstract concepts were categorized as ‘exteroceptive’ and concrete concepts as ‘interoceptive’ were considered as ‘alternative classifications’. We recognize that our task requires a dichotomous classification, but along a dimension that is inherently subjective rather than strictly objective. The complexity characterizing our task resembles that of the lexical decision task, where factors like list composition and the presence of ambiguous stimuli such as pseudowords influence responses (Barca & Pezzulo, 2012, 2015). The variability observed in alternative classifications may encompass a complexity that extends beyond a simple binary distinction. Rather than signifying errors, this variability reflects the subjective nature of conceptual processing. To acknowledge this inherent variability and avoid implying correctness or incorrectness, we have adopted the term'alternative classifications', particularly in contrast to the dominant classifications, which align with both frequency-based patterns and theoretical predictions.

As the ability to correctly perceive internal bodily signals (i.e., interoceptive accuracy) varies widely across individuals, the heartbeat counting task (Schandry, 1981) was employed to investigate whether it might influences conceptual representation. Despite some criticisms (Murphy et al., 2018; Windmann et al., 1999) it remains the most widely used measure of interoceptive accuracy, and for this reason, we have chosen to employ it.

Our primary hypothesis centered on the relationship between interoceptive accuracy and the categorization of abstract concepts. We predicted that individuals with higher interoceptive accuracy would exhibit faster and more frequent ‘interoceptive’ categorization of abstract concepts, particularly those with emotional content. Specifically, we expected the effect to be strongest for abstract-emotional concepts and moderate for abstract-philosophical concepts, reflecting the reliance of these categories on internal emotional and cognitive processing.

Furthermore, we examined the relationship between participants’ interoceptive sensibility, (the subjective self-reported awareness of internal bodily sensations, which reflects an individual's perceived ability to detect and interpret internal signals) and their emotional affective-related states and traits. Specifically, to characterize participants’ interoceptive sensibility and emotional-affective state, we administered self-report questionnaires, such as the Multidimensional Assessment of Interoceptive Awareness MAIA, (Calì et al., 2015; Mehling et al., 2012), which provides a measure of eight dimensions constituting interoceptive sensibility; and a series of questionnaires measuring generalized anxiety (Generalized Anxiety Disorder-7, GAD-7, Spitzer et al., 2006), depression (the Center for Epidemiologic Studies Depression Scale, CES-D, Radloff, 1977), and alexithymia (the Toronto Alexithymia Scale, TAS-20, Taylor et al., 1992).

## Methods

### Ethics statement

The procedure was approved by the Ethical Committee of Sapienza University of Rome (Protocol No. 0001106).

Written informed consent was obtained from the participants. Conflicts of interest: None.

### Participants

A sample of 40 female participants, aged 23–36 years (mean age 27.18), took part in the study. They were Italian speakers, right-handed, and with normal or corrected to normal vision. We excluded participants with cardiovascular, neurological, or psychiatric problems. The participants were all volunteers (they did not receive any payment for their participation), and the majority were recruited from the National Library of Rome. Due to biological sex-related differences in interoceptive abilities (both in terms of accuracy and sensibility, see also Barca et al., 2025; Grabauskaitė et al., 2017; Haruki et al., 2025; Naraindas et al., 2024; Prentice & Murphy, 2022) to reduce confounding effects due to the sample variability we decided to recruit only females.

### Materials

A list of 40 words was used as stimuli. The list included 20 concrete words (10 artifacts, 10 naturals) and 20 abstract words (10 emotional, 10 philosophical), taken from the database of Villani (Villani et al., 2019) and Della Rosa (Della Rosa et al., 2010). Concrete and abstract words were matched (all t-tests were not significant) for written frequency, familiarity, and length in letters.

### Ratings and self-report questionnaires

Participants rated the stimuli for abstractness, concreteness, Body Object Interaction (BOI, Pexman et al., 2019; Tillotson et al., 2008), emotionality, and perceived interoceptive ratings of concepts. Specifically, a series of visual-analogue scales (VAS), ranging from 0 to 100 points, were used for all ratings (see details in the Appendix). The ratings of interoception should be distinguished from the other measures of interoception (i.e., in our case Heartbeat Counting Task measuring interoceptive accuracy, and MAIA questionnaire measuring interoceptive sensibility), as they serve as an index of *conceptual knowledge* about interoception underlying different categories of abstract and concrete concepts, rather than a direct (behavioral or subjective) measure of interoceptive ability. We took the formulation of interoceptive ratings from previous work on English and Italian norms aimed at addressing the role of perceptual strength for concrete and abstract concepts. Ratings of interoception in English were collected and included in a large norming study on 39707 abstract and concrete concepts (Lynott et al., 2020). These norms include ratings on the five perceptual modalities, on five action effectors (mouth/throat, hand/arm, foot/leg, head excluding mouth/throat, and torso), and for the first time, ratings on what has been called “the forgotten modality”, i.e., interoception (Connell et al., 2018). The same formulation, translated and adapted to Italian, was used for the first time in a database including 15 semantic dimensions, including perceptual modalities, effectors, and inner dimensions that characterize abstract concepts (Villani et al., 2019), then employed in further studies and databases of Italian abstract and concrete concepts (e.g., Falcinelli et al., 2024; Mazzuca et al., 2022). Theoretically, introducing this semantic dimension broadens the scope of embodied and grounded cognition, according to which bodily experiences influence concept formation and use. Until some years ago, research on conceptualization mainly focused on sensorimotor experiences without considering the importance of the inner body. As previously highlighted, this dimension differs from the other construct related to interoception that we measure in this paper since it concerns semantics– it requires participants to scrutinize the meaning of the single words and verify whether they evoke inner bodily signals rather than focusing directly on their bodily experience. Participants also completed a series of self-report questionnaires to evaluate interoceptive sensibility (the subjective self-reported awareness of internal bodily sensations, which reflect an individual's perceived ability to detect and interpret internal signals).

The MAIA (Mehling et al., 2012) was employed for assessing interoceptive sensibility. The questionnaire consists of 32 items, which assess eight distinct facets of interoceptive sensibility: Noticing (awareness of bodily sensations), Not-Distracting (tendency to avoid distraction or ignore painful or uncomfortable sensations), Not-Worrying (resilience to emotional distress caused by uncomfortable sensations), Attention Regulation (ability to focus attention on and control body sensations), Emotional Awareness (recognition of the relationship between emotions and bodily sensations), Self-Regulation (management of distress through attention to bodily sensations), Body Listening (deliberate attention to insight from the body), and Trusting (confidence and safety in bodily experiences). Higher scores indicate greater sensibility to bodily signals. We utilized the Italian version of the MAIA, the validation of which for the Italian language has been detailed by Calì and colleagues (Calì et al., 2015).

The GAD-7 (Generalized Anxiety Disorder-7, Spitzer et al., 2006) is designed to assess the severity of generalized anxiety disorder symptoms in adults. It consists of seven items that inquire about various symptoms commonly associated with generalized anxiety disorder, such as feeling nervous, worrying excessively, and experiencing difficulty relaxing. Each item is rated on a scale from 0 to 3, with higher scores indicating more severe symptoms. The total score ranges from 0 to 21, with higher scores indicating a higher level of anxiety. The GAD-7 has been widely used in both clinical and research settings as a brief and reliable tool for assessing generalized anxiety disorder symptoms.

The TAS-20 (Toronto Alexithymia Scale-20 items, Taylor et al., 1992); (Bressi et al., 1996 for the Italian validation) has been used to measure the presence of an alexithymic profile. The questionnaire identifies three separates, yet conceptually related, facets of the alexithymia construct: i) difficulty identifying feelings and distinguishing them from the somatic sensations that accompany emotional arousal, ii) difficulty communicating feelings to other people, and iii) externally oriented thinking.

The CES-D, or Center for Epidemiologic Studies Depression Scale, is designed to measure depressive symptoms in the general population. Developed by Radloff in 1977, we have used the Italian validation (Fava, 1983). The CES-D consists of 20 items that assess various symptoms commonly associated with depression, such as feelings of sadness, hopelessness, and loss of interest in activities. The respondents indicate how often they experienced each symptom over the past week on a scale ranging from 0 (rarely or none of the time) to 3 (most or all of the time). Total scores can range from 0 to 60, with higher scores indicating more severe depressive symptoms. The CES-D is frequently used in research studies and clinical settings to screen for depression and monitor changes in depressive symptoms over time.

### Experimental session

Upon their arrival, the participants provided written informed consent to participate in the study and processing of the data.

They then performed the *Heartbeat Counting task* (HBCT) to measure cardiac interoceptive accuracy (IAc). We chose to use HBCT as it is the most widely employed task in the cardiac domain. However, we acknowledge that heart rate perception can be influenced by various physiological and psychological factors (Murphy et al., 2018), including personal beliefs (Rouse et al., 1988; Zamariola et al., 2018), exteroceptive cues and strategies (Desmedt et al., 2018, 2023), heart rate variability (Knapp-Kline & Kline, 2005), body fat percentage (Rouse et al., 1988), and systolic blood pressure (O’Brien et al., 1998). To minimize the influence of prior beliefs, exteroceptive cues, and cognitive strategies, we provided participants with clear and precise instructions, explicitly guiding them to report only the heartbeats they genuinely perceived, rather than making random guesses (see Desmedt et al., 2018 for a similar point).

The task was performed using four intervals of 25 s, 35 s, 45 s, and 100 s duration (Pollatos et al., 2008; Schandry, 1981), presented in randomized order. During all trials, the participants were asked to silently count their heartbeats. A start and stop auditory cue signaled the beginning and end of the counting phases. The participants were not allowed to take their pulse or attempt to use other forms of manipulation to aid in counting their heartbeats. Following the stop signal, participants were required to type the number of counted heartbeats. During the task, the participants were connected to a portable ECG unit (MyHeart) sampling at 1000 Hz with two single-use Ag/AgCl electrodes. A Matlab custom script (The MathWorks, Inc.) was used to identify and count the number of R-wave peaks on the ECG trace, which was also visually inspected for artifacts.

The number of reported heartbeats was compared with the real number of heartbeats measured. The heartbeat perception score reflecting the accurate perception of the interoceptive cardiac signal was calculated as the mean score of four heartbeat perception intervals according to the following transformation:

- FORMULA: 1/4 Σ (1 − (|recorded heartbeats − counted heartbeats|/recorded heartbeats)).

IAc scores can vary between 0 and 1, where lower values indicate poorer performance.

Next, after a short break, they performed the *Interoception-Exteroception judgment task* with the mouse tracker software (Freeman & Ambady, 2010), allowing us to collect kinematic measures of decision-making in both time (response times) and space (movement patterns and accuracy). The participants were presented with the following instructions on the computer screen: “*Your task is to categorize a series of stimuli, namely words, as belonging to interoception or exteroception. Indeed, words can be experienced through sensations within the body (e.g., you feel your heart beating, your breathing changing, your stomach contracting or rumbling, your internal temperature changing) or through the five senses (e.g., touching, smelling, observing, tasting, and listening). We will present you with a series of words appearing in the center of the screen, and your task will be to indicate whether you experience each word through sensations within the body or through the five senses. If you experience it through sensations inside the body, move the mouse and press the top left button ('INTERNAL'), if you experience it with the five senses, move the mouse and press the top right button ('EXTERNAL'). Try to be quick in answering*.’

After reading the instructions and a short practice, the participants started the task. At the beginning of each trial, the participants were instructed to click on the/START/button located at the bottom center of the screen. A central fixation cross appeared and (after 300 ms) was replaced by a stimulus, i.e. the concrete or abstract word (visible for 500 ms). The participants had two seconds to respond; otherwise, a time-out feedback appeared. Regardless of where they clicked within the START button, the software automatically relocated the mouse to the origin of the Start button (i.e., the cursor is repositioned to the center of the button). Each trial lasted a maximum of 2800 ms (from when the participant pressed/START/until their response), and the overall mouse tracker task lasted approximately 7 min. The position of the responses was counterbalanced so that 23 participants responded with Internal on the left top corner of the screen and External on the right (script A), and 24 participants responded according to the opposite mapping (i.e., External on the left, Internal on the right - script B). The experimental list of 40 stimuli was presented twice – in two blocks with a break in between - to increase the number of observations. The order of the stimuli within each block was randomized by the software. The experimental data collection was preceded by a brief practice session with 6 (non-experimental) stimuli.

The experimental session ended with a debriefing with the participant and with the sending of the link to complete the self-report questionnaires (MAIA, CES-D, GAD-7, TAS) and ratings of the stimuli (abstractness, concreteness, BOI, emotionality, interoceptive ratings) at home via Qualtrics.

### Mouse tracker data of the interoceptive-exteroceptive judgment task

Response time (RT) and categorization rate were collected, with parameters measuring mouse movement in the spatial domain (i.e., trajectories Maximum Deviation – MD - and Area Under the Curve - AUC).

Participants’ response time (RT) in milliseconds was recorded from the time they clicked/START/to the time they clicked on the selected response option (Exteroception/Interoception). Mouse trajectory x–y coordinates were automatically recorded by the software, indexing the location of the mouse along the horizontal and vertical axes.

The measure of Maximum Deviation (MD), calculated from the mouse coordinates, is defined as the most substantial perpendicular deviation between the actual mouse trajectory and the ideal mouse trajectory (a straight line from the/START/to the endpoint of the trajectory). Higher MD scores reflect participants’ attraction to the unselected choice.

The Area Under the Curve (AUC) is the space under the curve created by the actual trajectory in comparison to the ideal trajectory. Higher scores reflect participants’ overall attraction to the unselected choice across all time steps.

### Data processing and statistical analysis

In the Interoceptive-Exteroceptive Judgment task, ‘dominant responses’ refer to the categorizations that received the highest frequency among participants for each concept. Importantly, these dominant responses also align with our a priori hypotheses: abstract concepts were expected to be classified as ‘interoceptive’, while concrete concepts were expected to be classified as ‘exteroceptive’. Therefore, dominant responses represent both the most frequent categorizations and those consistent with our theoretical predictions. In contrast, alternative responses, those deviating from the dominant categorizations, were analyzed to investigate variability in conceptual processing.

Alternative responses were removed from the response time data analysis (see below). This decision was based on both theoretical and kinematic considerations within the categorization task, which led us to analyze only the majority-dominant responses for each concept. Theoretically, alternative responses (e.g., classifying a concrete artifact concept as ‘interoceptive’) suggest a fundamentally different conceptualization process, potentially indicating unique categorization criteria or varying conceptualizations of the categories among participants. Kinematically, these alternative responses involve movement trajectories that deviate significantly from those associated with the dominat responses for a given category, implying a distinct motor planning process. For instance, with a left-for-interoceptive and right-for-exteroceptive mapping, an alternative response entails movement in the opposite direction. This is particularly relevant for our right-handed participants, as movements towards the right (ipsilateral) are typically faster and more precise than those towards the left (contralateral). Analyzing alternative responses alongside the dominant responses could therefore confound the data, as the motor component might be influenced by both the cognitive categorization and biomechanical factors related to hand dominance and movement direction. While we acknowledge the limitations of focusing solely on dominant responses, we believe this separation is crucial for a clearer understanding of the distinct cognitive and motor processes underlying each type of response. To mitigate potential bias related to movement direction, response positions were counterbalanced across participants.

The response time outliers were detected and removed with the R code ‘outlierKD’, which uses Tukey’s method to identify the outliers ranging above and below the 1.5 interquartile range (Diachkov, 2024).

Linear mixed-effects models (LMM) were used to analyze Response Time of dominant responses and the other indexes of the trajectories. To do that we used the ‘lmerTest’ package in R4.1.0 (Kuznetsova et al., 2017). LMM tested for an effect of Stimulus Category (concrete-artifact, concrete-natural, abstract-emotional, abstract-philosophical) and cardiac interoceptive accuracy (IAc) while accounting for Trial and Subject variances. A Likelihood ratio test was used to compare different models varied for the complexity of the random structure. Once the best model has been established, the ANOVA tested the significance of the fixed factors within the mixed model of different outcome variables (see Supplementary Materials for details). The ‘ls_means’ function compared the pairwise differences between the levels of Stimulus Category. When appropriate, the ‘test interaction’ of the Phia package was used to test if the effect of IAc on the outcome variables varied across different levels of the Stimulus Category (that is, testing if the effect of IAc was homogeneous between different stimuli). Furthermore, where appropriate, Gardner-Altman estimation plots were used to visualize Cohen's d and its 95% confidence intervals, providing an assessment of effect sizes beyond traditional p-values (Ho et al., 2019). A generalized linear mixed-effects model (GLMM) was used to assess the impact of Stimulus Category, IAc and interoceptive ratings on classification rates (Baayen et al., 2008; Bolker et al., 2009). GLMM fit by maximum likelihood (Laplace Approximation) was implemented in R with the ‘lme4’ package (Bates et al., 2011; Jaeger, 2008). The binomial family was used to model the categorical classification responses, where'0'represented the dominant classification (i.e., the response given by the majority of participants for a given stimulus), and'1'represented the alternative response. The model included a random intercept for Subjects and Items, with a maximal by-subject random structure as a baseline model (Barr et al., 2013). A Likelihood ratio test was used to compare different models varied for the complexity of the random effects structure (see Supplementary Materials for details). The ‘Emmeans’ package (Lenth, 2017) was used to test significant interactions.

## Results

### Participant characteristics

#### Interoceptive accuracy (IAc)

The analysis was performed on 38 out of 40 participants (one participant did not complete the task, and the data of another participant were discarded as the signal was damaged due to a malfunction of the device).

The mean score of the sample was 0.44 (sd = ± 0.23), and approximately 53% of the participants had an IAc score below 0.50, so the majority of the sample did not have good interoceptive accuracy (as measured with this task). A minority of the sample (10%) had a score greater than 0.70, suggesting good accuracy in perceiving the cardiac signal.

#### Participants’ self-reported measures of affectivity

Considering the different facets of interoceptive sensitivity as measured by the MAIA (Mehling et al., 2012), participants had the highest score on the Noticing subscale (mean = 3.64; sd = ± 0.74), a measure of interoceptive sensibility, which might reflect maladaptive aspects of attention to bodily sensation (Mehling, 2016). High scores on Emotional Awareness (mean = 3.41; sd = ± 0.97) suggest good self-reported awareness of bodily sensations. However, if this awareness is not accompanied by the ability to regulate these emotions to reduce discomfort (here indicated by a low score of 2.08 on Self-Regulation), it could also contribute to an increase in anxious states (Mehling et al., 2012).

Participants had a rather low score on Not-Worrying (mean = 1.97; sd = ± 0.84) suggesting reduced resilience to emotional distress caused by uncomfortable sensations. The scores on the other subscales were in the middle of the scale, with Attention Regulation and Body Listening (mean = 2.42; sd = ± 0.79 and mean = 2.47; sd = ± 1.13 respectively), reflecting the ability to sustain and control attention to bodily sensations and actively listen to the body for insight; Not-Distracting and Trusting (mean = 2.68; sd = ± 0.71 and mean = 2.80; sd = ± 1.28 respectively) reflecting the tendency not to ignore uncomfortable sensations and a sense of trust in one’s own body.

With respect to the TAS questionnaire, individuals with high scores may have difficulty in recognizing their own emotions and distinguishing them from bodily sensations. The mean score of the sample (47.2, sd = ± 16) is within the cut-off of 51 for the Italian population (Caretti et al., 2005). However, 16% of our sample had a borderline score (between 51 and 60), and 21% scored higher than 61, which is considered an indication of possible alexithymia. Considering the subtype score of the TAS, the highest score was on the third subscale - externally oriented thinking (mean of 17.2, sd = ± 5.3) - and the first subscale - difficulties identifying feelings (mean score of 16.5, sd = ± 6.4). The second subscale, difficulties describing feelings, had a mean score of 12.9 (sd = ± 6.5).

Concerning the screening for anxiety (Spitzer et al., 2006), the sample had an overall mean score of 9.8 (sd = ± 4.8). Thirteen percent of the sample had a score suggesting minimal anxiety (score 0–4), 31% had a score suggesting mild anxiety (score 5–9), 37% had moderate anxiety (score 10 to 14), and 19% had a possible severe level of anxiety (from 15–21).

For the CES-D, screening for depression, the overall mean score of the sample was rather high (25.2, sd = ± 7.4), considering the cut-off value of 16 provided by Fava (Fava, 1983) for the Italian population. Specifically, the score of 22% of the sample suggests moderate symptoms of depression, but for 50% of the participants, the presence of more severe symptoms associated with major depression is suggested (see also Radloff, 1977).

To summarize, most of the study samples had poor cardiac interoceptive accuracy. Self-reported measures of interoceptive awareness (MAIA scores) suggest great attention to bodily sensations. From an affectivity standpoint, elevated levels of alexithymia are observed (particularly a tendency toward externally oriented thinking and difficulty in recognizing emotions), with medium to high levels of anxiety and depression.

### Subjective ratings of the conceptual categories of the stimuli

Table 1 summarizes the descriptive statistics (means and standard deviations) for abstractness, concreteness, Body-Object interaction, emotionality and interoceptive ratings across different categories.

**Table 1:** Means and standard deviations (in brackets) for abstractness (ABS), concreteness (CONC), interoception (INTERO), emotionality (EMO), and body object interaction (BOI) across different stimulus category: abstract-emotional (abs-emo), abstract-philosophical (abs-phr), concrete-artifact (con-art), concrete-natural (con-nat)

Stimulus Category	ABS	CONC	INTERO	EMO	BOI
abs-emo	51.1 (8.52)	46.22 (7.3)	57.17 (6.7)	57.32 (9.01)	44.96 (8.46)
abs-phr	64.92 (13.56)	36.7 (11.34)	40.93 (12.33)	45.62 (14.72)	33.06 (15.32)
con-art	9.97 (6.4)	83.69 (5.55)	13.05 (4.63)	13.95 (6.62)	69.96 (13.64)
con-nat	12.81 (4.8)	78.87 (5.63)	14.97 (9.38)	19.91 (13.32)	67.07 (6.84)

Abstract-philosophical concepts exhibit the highest mean abstractness rating (64.92), while concrete-natural concepts show the lowest (9.97). Conversely, concrete-artifact concepts present the highest mean concreteness rating (83.69), while the abstract-philosophical ones have the lowest (36.7). This aligns with the abstractness findings, indicating an inverse relationship between abstractness and concreteness.

Abstract-emotional concepts show the highest mean interoceptive rating (57.17), while concrete natural and artifact concepts have the lowest (13.05 and 14.9, respectively). Similar to interoceptive ratings, the abstract-emotional concepts exhibit the highest mean emotionality rating (57.32), indicating that they were perceived as more emotionally arousing.

Finally, concrete artifact and natural concepts show the highest mean BOI ratings (69.96 and 67.07, respectively), while abstract-philosophical has the lowest (33.06). This pattern suggests that stimuli of the concrete category elicited a stronger sense of body-object interaction.

To further explore the relationships between these variables, we conducted Pearson's correlations, the results of which are presented in Table 2. Pearson's correlation coefficients were calculated using R (R Core Team, 2023) with the ‘correlation’ package. To account for multiple comparisons, we applied the Holm (1979) method for *p*-value adjustment.

**Table 2:** Pearson's correlations between subjective ratings of abstractness (ABS), concreteness (CONC), interoceptive ratings (INTERO), emotionality (EMO), and body-object interaction (BOI)

	BOI	EMO	INTERO	CONC
ABS	−0.76***	0.80***	0.74***	−0.98***
CONC	0.77***	−0.83***	−0.77***	
INTERO	−0.69***	0.89***		
EMO	−0.59***			

Asterisks indicate the level of statistical significance (*p* < 0.05, ** *p* < 0.01, and *** *p* < 0.001)

Focusing on interoceptive ratings, a positive correlation was found between interoceptive and abstractness ratings indicating that stimuli perceived as more abstract tend to be associated with higher interoceptive ratings. Conversely, the negative correlation between interoceptive and concreteness ratings suggests that the more stimuli are categorized as concrete, the lower they are categorized as interoceptive. The positive correlation between interoceptive ratings and emotionality indicates that stimuli rated high in emotionality tend to be associated with higher interoceptive ratings. Finally, the moderate negative correlation with BOI suggests that stimuli that elicit a stronger sense of body-object interaction tend to be associated with lower interoceptive ratings.

**Fig. 1:**
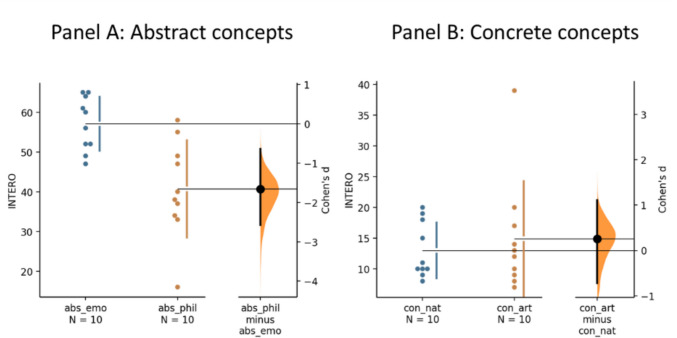
Estimation plots for interoceptive rating differences between stimulus category: abstract-emotional (abs-emo), abstract-philosophical (abs-phr), concrete-artifact (con-art), concrete-natural (con-nat). The Cohen's d between abstract emotional and philosophical concepts (Panel **A**) and concrete natural and artifact concepts (Panel **B**) is shown in the Gardner-Altman estimation plot. Groups are plotted on the left axes; the mean difference is plotted on a floating axis on the right as a bootstrap sampling distribution. The mean difference is depicted as a dot; the 95% confidence interval is indicated by the ends of the vertical error bar

To examine differences in interoceptive ratings across stimulus categories, we employed estimation graphics (Fig. 1), an approach that emphasizes effect sizes and confidence intervals over traditional p-values (Ho et al., 2019).

The unpaired Cohen's d between interoceptive ratings of emotional and philosophical abstract concepts (panel A) is −1.66 [95.0%CI −2.57, −0.639], with the p value of the two-sided permutation t-test equal to 0.001. The large Cohen's d and its confidence interval suggest a significant difference. As for the concrete category, the Cohen's d between interoceptive ratings of natural and artifact concepts is equal to 0.258 [95.0%CI −0.72, 1.11], with *p* value = 0.649 of the two-sided permutation. Panel B shows a lack of a meaningful or statistically significant effect, suggesting that concrete natural and artifact concepts do not differ on interoceptive ratings (i.e., conceptual knowledge of interoception).

### Interoceptive-exteroceptive judgment task

The sample comprises 38 participants. Overall, 19% of alternative responses were made—that is, cases in which the interoceptive/exteroceptive response differed from our classification—and the other 4% of cases were out-of-time responses (exceeding the time limit). The dominant response pattern across participants was consistent with our classification of abstract concepts as ‘interoceptive’ and concrete concepts as ‘exteroceptive’. However, a minority of responses deviated from this pattern. Specifically, abstract-philosophical concepts were classified as exteroceptive in 27% of cases, concrete-natural concepts as interoceptive in 20% of cases, abstract-emotional concepts as exteroceptive in 17% of cases, and concrete-artifact concepts as interoceptive in 12% of cases.

#### Analysis of dominant responses, in the temporal domain (response time, movement initiation time) and spatial domain (area under the curve, trajectories’ maximum deviation)

**Fig. 2:**
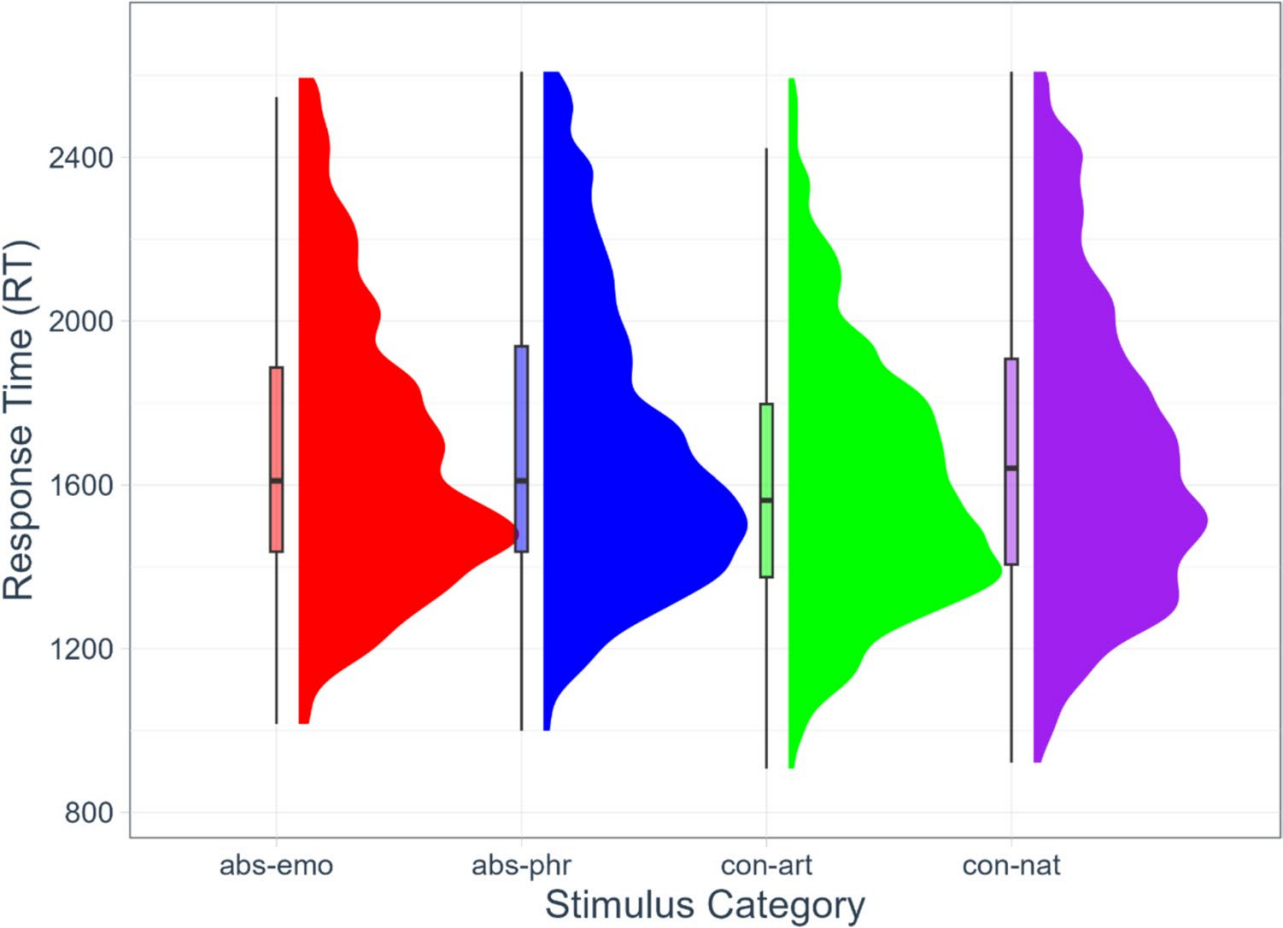
Distribution of reaction times (RTs) by stimulus category: abstract emotional (abs-emo), abstract philosophical (abs-phr), concrete artifact (con-art), concrete natural (con-nat). Graphs show data distribution (half-eye) and boxplots for each category. The half-eye represents the probability density of the data, while boxplot shows the median, quartiles, and outliers. Stimulus categories are represented on the x-axis, and reaction times (RTs) on the y-axis

The trimming of the response time distribution (OutlierKD in R) identified and removed 31 observations because they were outliers. Figure 2 shows the reaction times of the dominant categorizations based on the Stimulus Category, where the violin represents the probability density (half-eye visualization) of RT values within each concept category, and the boxplot displays the median and inner-quartile range. The concrete-artifact concepts had a mean response time of 1602 ms (sd = 308), the abstract-philosophical concepts had a response time of 1698 ms (sd = 348), the abstract-emotional concepts had a response time of 1680 ms (sd = 340), and the concrete-natural concepts had a response time of 1678 ms (sd = 363).

We constructed a linear mixed model (LMM) with Response Time as the dependent variable. Specifically, we used the lmerTest package in R to fit our LMMs. This package provides methods for calculating degrees of freedom and conducting hypothesis tests that are more appropriate for LMMs than relying on the asymptotic chi-squared approximation (for a complete description of the model selection, including ANOVA and BIC comparisons, please refer to the Supplementary Materials). The final model adopted includes the interaction between Stimulus Category and Interoceptive Accuracy (IAc), with random intercepts for subjects and trials. Specifically, it can be represented as: RT ~ Stimulus Category * IAc + (1 | subject) + (1 | trial). Detailed of the LMMs output can be found in the Supplementary Materials.

The MuMIn R package (Bartoń, 2010) was used to compute a PseudoR2, which indicates that our statistical model explained 59% of the variance with a large effect size (f2 = 1.5) according to Cohen’s conceptualization (Cohen, 1988).

The linear mixed-effects model, with reaction times (RTs) as the dependent variable, demonstrated a good fit to the data (REML criterion at convergence = 32079.2). The random effects section revealed significant inter-subject variability, indicating that individual differences among subjects substantially influenced the observed reaction times (variance of random intercept of'subject'= 52362, stdev = 228.83). The random intercept for'trial'also contributed to the model (variance = 8888, stdev = 94.28).

The results of the Type III Analysis of Variance with Satterthwaite's method for the lmerTest model are presented below. The ANOVA table on *Response Time* indicates a main effect of Stimulus Category (F (3, 2209.1) = 9.20, *p* = 4.757e-06, partial η^2^ = 0.0123) and its interaction with IAc (F (3, 2209.2) = 2.68, *p* = 0.045, partial η^2^ = 0.0036). IAc did not affect response times per se (F (1, 35.7) = 0.71, *p* = 0.406, partial η^2^ = 0.0003). The pairwise difference of Least Squares Means on Stimulus Category indicates no difference within the abstract category (Estimates _Emo-Phil_ = −12.5, SE = 15.77, df = 2224.0, *t* value = −0.79, lower = −43.42, upper = 18.44, *p* = 0.428), and faster responses for concrete-artifact than concrete-natural stimuli (Estimates _Art-Nat_ = −85.3, SE = 15.09, df = 2233.6, *t* value −5.65, lower = −114.88, upper = −55.69, *p* = 1.789e-08). For the between-category differences, abstract-emotional concepts did not differ from concrete-natural concepts (Estimates _Emo-Nat_ = −24, SE = 15.36, df = 2224.9, *t* value = −1.56, lower = −54.130, upper = 6.126, *p* = 0.118) but were slower than concrete-artifact concepts (Estimates _Emo-Art_ = 61.3, SE = 15.006, df = 2234.6, *t* value = 4.08, lower = 31.862, upper = 90.715, *p* = 4.576e-05). Abstract-philosophical concepts did not differ from concrete-natural concepts (Estimates _Phil-Nat_ = −11.5, SE = 16.178, df = 2237.2, *t* value = −0.712, lower = −43.238, upper = 20.212, *p* = 0.4758) but were slower than concrete-artifact (Estimates _Phil-Art_ = 73.8, SE = 15.688, df = 2233.3, *t* value = 4.7, lower = 43.012, upper = 104.542, *p* = 2.724e-06).

**Fig. 3:**
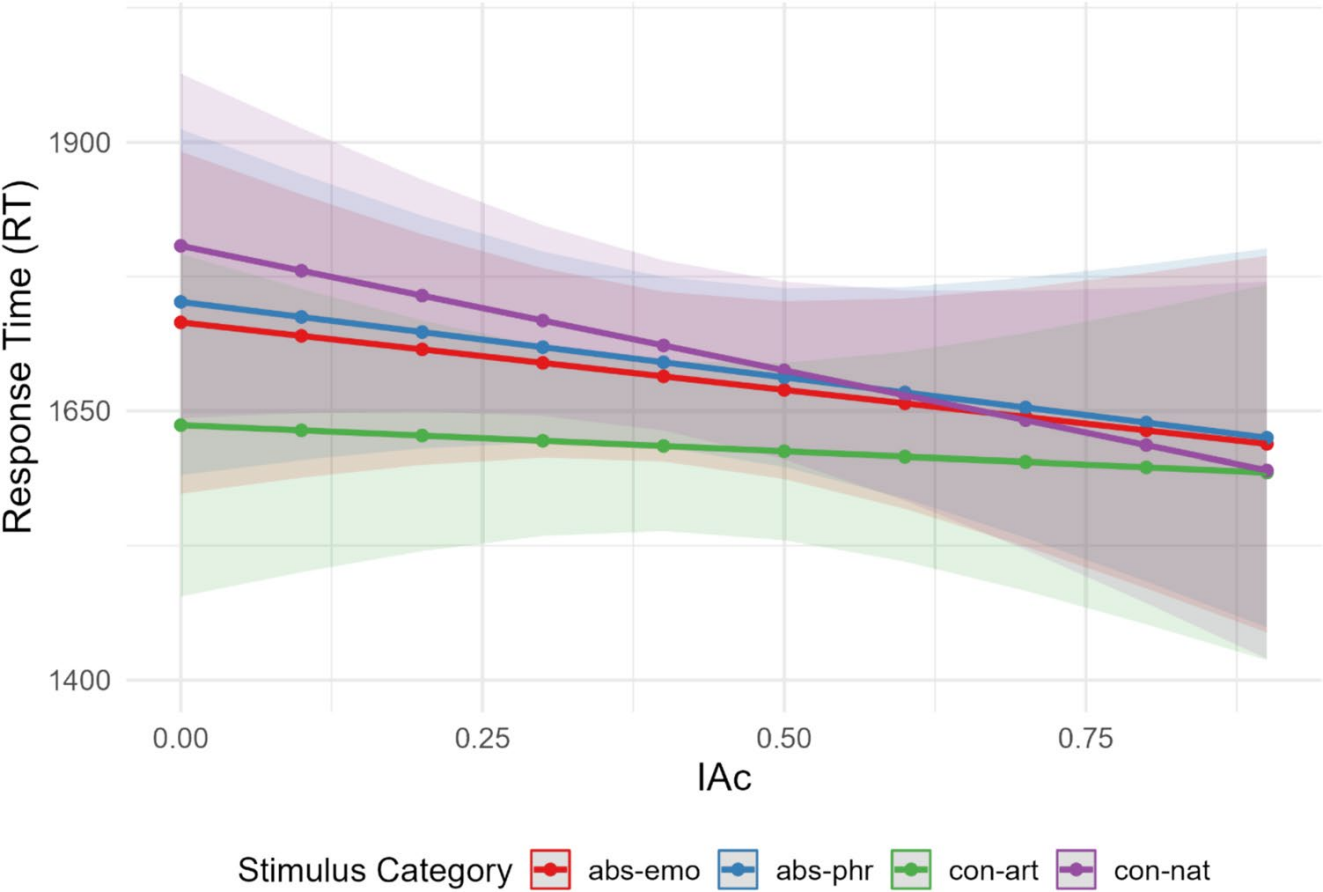
Predicted response times (RT) with 95% confidence intervals as a function of IAc and stimulus category: abstract emotional (abs-emo), abstract philosophical (abs-phr), concrete artifact (con-art), concrete natural (con-nat)

With respect to the Stimulus Category*IAc interaction, see Fig. 3 plotting the values of RT predicted by the Stimulus Category and IAc interaction.

Inspection of Fig. 3 suggests that higher IAc scores reduce the response time for dominant categorization, with a modulation of the facilitation effect according to Stimulus Category (i.e., more marked for concrete-natural concepts, more reduced for concrete-artifact concepts). The post hoc test of interaction (pairwise differences in the stimulus category with slope IAc, p-adjusted with the Holmes method) indicates that the effect of IAc is not homogenous across the different stimuli and is significant only within the concrete category (Value _Art-Nat_ = 183, SE = 64.71, Chi square = 8, *p* = 0.028).

The movement *Initiation Time* was not affected by the Stimulus Category (*F*(3, 2281.31) = 0.97, *p* = 0.405, partial η^2^ = 0.0013.), IAc (*F*(1, 35.45) = 0.83, *p* = 0.369, partial η^2^ = 0.0004), or their interactions (*F*(3, 2279.14) = 1.08, *p* = 0.358, partial η^2^ = 0.0014), indicating that the effect of Stimulus Category on the initiation of movement response did not differ significantly depending on IAc.

**Fig. 4:**
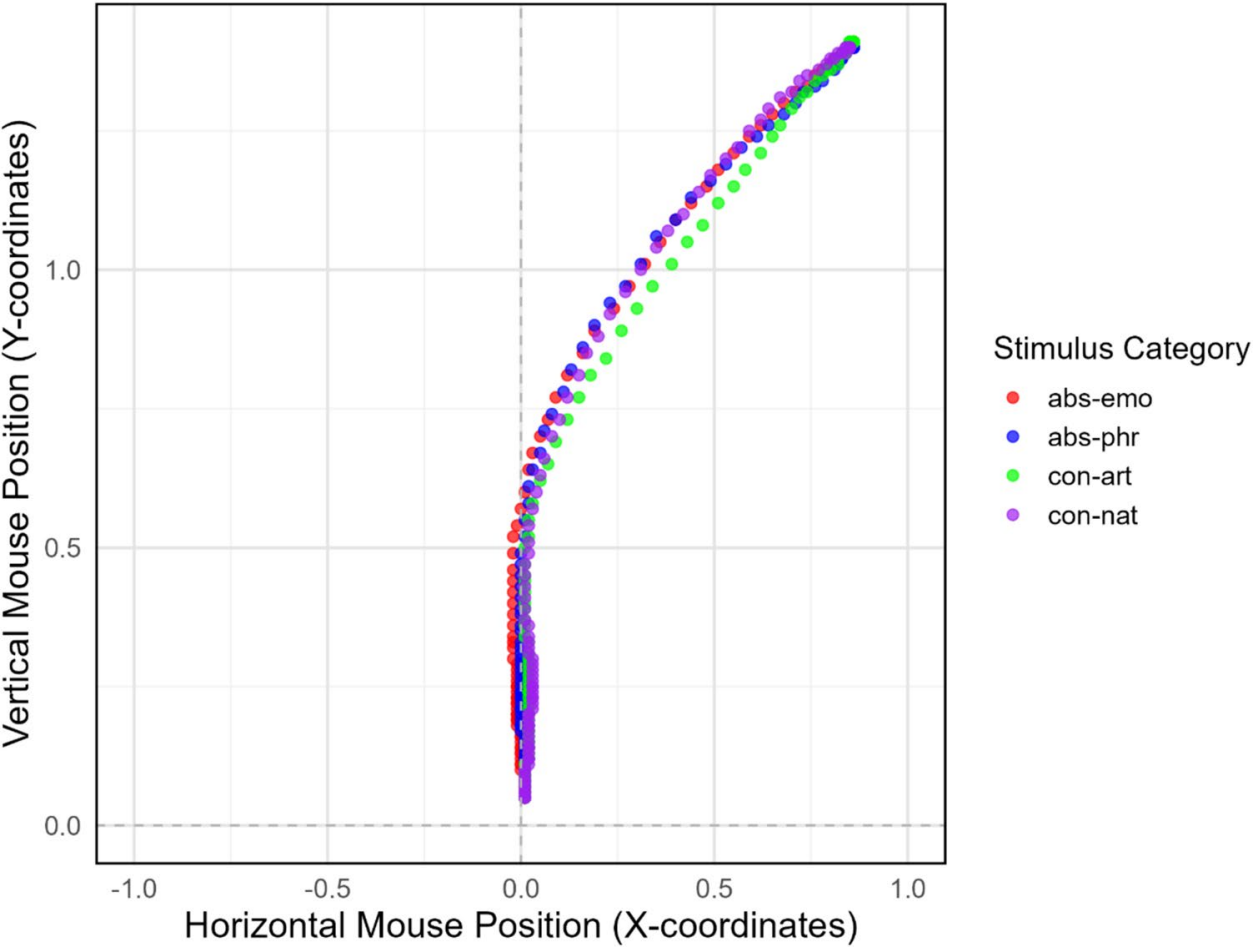
Average mouse trajectories by stimulus category, rightward remapped. The plot displays the horizontal and vertical coordinates of mouse movements, with each color indicating a different Stimulus Category: abstract emotional (abs-emo), abstract philosophical (abs-phr), concrete artifact (con-art), concrete natural (con-nat)

Considering movement trajectories, Fig. 4 shows the average of the correct response trajectories per stimulus category.

Figure 4 shows the overall similarity within the abstract emotional and philosophical concepts category (red and green dots, respectively). Compared with concrete-natural concepts (purple dots), concrete-artifact concepts (blue dots) have straighter response trajectories than the other categories do.

The analysis of the *AUC values* revealed a main effect of Stimulus Category (*F* (3, 2309.2) = 3.18, *p* = 0.023, partial η^2^ = 0.0041) indicating that, on average, there were significant differences in AUC values across the different concepts. IAc was not significant (*F* (1, 2263.6) = 0.00, *p* = 0.998, partial η^2^ ≈ 0.00) nor was the interaction (*F* (3, 2309.9) = 0.91, *p* = 0.436, partial η^2^ = 0.0012). Pairwise comparisons of Least Squares Means on the Stimulus Category indicate no difference in AUC values within the abstract category (Estimates _Emo-Phil_ = −0.002, SE = 0.0596, df = 2257.5, *t* value = −0.0424, lower = −0.11945, upper = 0.1144, *p* value = 0.9662), and a difference within the concrete category (Estimates _Art-Nat_ = −0.16, SE = 0.0569, df = 2186.5, *t* value = −2.8209, lower = −0.2725, upper = −0.0490, *p* = 0.0048) with smaller AUC values for artifact than natural concepts. For the between-category differences, abstract-emotional concepts did not differ from concrete-natural concepts (Estimates _Emo-Nat_ = 0.02, SE = 0.0580, df = 2255.4, *t* value = 0.3631, lower = −0.0926, upper = 0.1348, *p* value = 0.7165) but have greater AUC values than concrete-artifact (Estimates _Emo-Art_ = 0.18, SE = 0.0564, df = 2168.0, *t* value = 3.22, lower = 0.0710, upper = 0.2926, *p* value = 0.0013). Abstract-philosophical concepts did not differ from concrete-natural concepts (Estimates _Phil-Nat_ = 0.02, SE = 0.0601, df = 2172.6, *t* value = 0.3920, lower = −0.0944, upper = 0.1416, *p* value = 0.6950)) but had greater AUC values than concrete-artifact did (Estimates _Phil-Art_ = 0.18, SE = 0.0586, df = 2209.7, *t* value = 3.14, lower = 0.0694, upper = 0.2993, *p* value = 0.002).

A similar pattern emerged for the trajectories’ *Maximum Deviation.* The main effect of Stimulus Category was statistically significant (*F* (3, 2310.0) = 4.01, *p* = 0.007, partial η^2^ = 0.0052), indicating that, on average, there were significant differences in MD values across the different concepts. The main effect of IAC was not statistically significant (*F* (1, 2263.6) = 0.0011, *p* = 0.973, partial η^2^ ≈ 0.00) nor the interaction (*F* (3, 2310.7) = 1.32, *p* = 0.266, partial η^2^ = 0.0017). Pairwise comparisons of Least Squares Means on the Stimulus Category indicate no difference in MD values within the abstract category (Estimates _Emo-Phil_ = 0.02, SE = 0.0596, df = 2261.8, *t* value = 0.39, lower = −0.0933, upper = 0.1404, *p* = 0.6922), and a difference within the concrete category (Estimates _Art-Nat_ = −0.12, SE = 0.0569, df = 2139.7, *t* value = −2.16, lower = −0.2346, upper = −0.0112, *p* = 0.031) with smaller MD values for artifact than for natural concepts. As for the between-category differences, abstract-emotional concepts did not differ from concrete-natural concepts (Estimates _Emo-Nat_ = 0.08, SE = 0.057, df = 2259.7, *t* value = 1.475, lower = −0.028, upper = 0.199, *p* = 0.140) but had greater MD values than concrete-artifact concepts did (Estimates _Emo-Art_ = 0.21, SE = 0.056, df = 2176.1, *t* value = 3.69, lower = 0.0977, upper = 0.3192, *p* = 0.0002). Abstract-philosophical concepts did not differ from concrete-natural concepts (Estimates _Phil-Nat_ = 0.06, SE = 0.0602, df = 2180.4, *t* value = 1.03, lower = −0.056, upper = 0.1799, *p* = 0.303) but had greater MD values than concrete-artifact concepts did (Estimates _Phil-Art_ = 0.18, SE = 0.0586, df = 2216.1, *t* value = 3.154, lower = 0.0699, upper = 0.2998, *p* = 0016).

To summarize, indices of movement trajectories indicate that both abstract-emotional and abstract-philosophical concepts were more attracted by the competing category than were concrete artifact concepts, nor natural ones.

#### Analysis of categorization rates

**Fig. 5:**
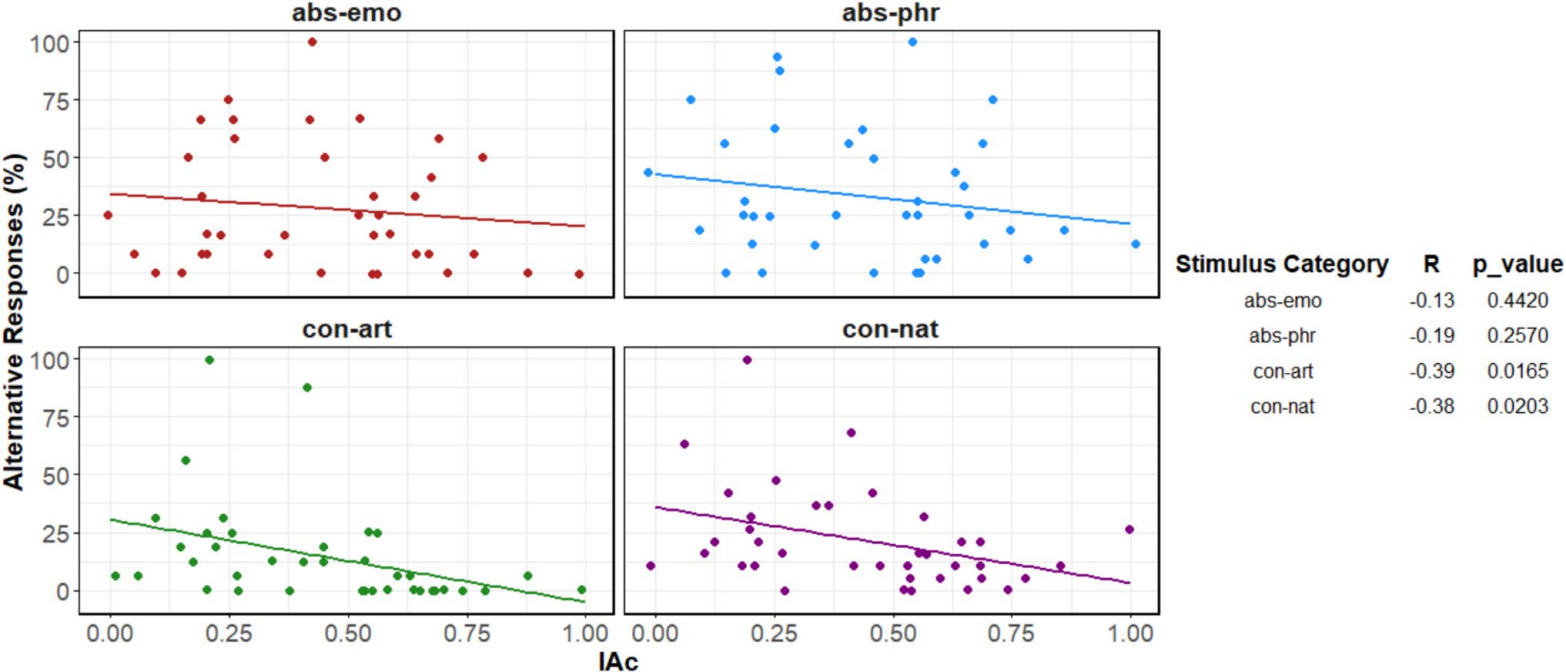
Correlations between percentages of alternative responses and interoceptive accuracy (IAc) across stimulus category: abstract emotional (abs-emo), abstract philosophical (abs-phr), concrete artifact (con-art), concrete natural (con-nat)

By “alternative responses,” we mean cases where abstract concepts (emotional and philosophical) have been categorized as ‘exteroceptive,’ and concrete concepts (artifacts and natural) have been classified as ‘interoceptive.’ The scatterplots in Fig. 5 show the relationships between alternative responses and IAc, across the different stimulus categories.

The plots display a negative slope between interoceptive accuracy and alternative responses for concrete concepts (both artifacts and natural), such that as IAc increases, the number of alternative responses for this category significantly decreases. A negative relationship was also present for abstract concepts (both emotional and philosophical), but it did not reach significance, possibly due to greater variability.

Some outliers were also present in cases of concrete-artifact and concrete-natural concepts or in participants who classified more than 75% of the trials of concrete natural concepts as ‘interoceptive’. Although these may not necessarily be considered errors, it is nevertheless a percentage of alternative responses that deviates from the trend of the rest of the group. These participants had very low values of interoceptive accuracy. Specifically, two participants for abstract philosophical concepts (subject 18, IAc = 0.50, 80% alternative responses; subject 46, IAc = 0.25, 75% alternative responses) and one for the concrete concepts (subject 47, IAc = 0.18, with 80% and 95% alternative responses of artifacts and natural concepts, respectively) were included.

Categorization rates were analyzed using a generalized linear mixed-effects model (GLMM) with a binomial distribution, modeling the binary response data (0 = dominant response, 1 = alternative response). The model included fixed effects for Stimulus Category (categorical), Interoceptive Accuracy (IAc, continuous), interoceptive ratings (continuous), and their interaction. The model also incorporated a random intercept for subject. The model was fitted using maximum likelihood with Laplace approximation and the'bobyqa'optimizer (details of the model fitting process and summary output are available in the Supplementary Materials).

The model demonstrates a reasonable fit, as evidenced by the AIC and BIC values, which balance model fit with complexity. The random effects section highlights significant inter-subject variability, with a variance of 0.603 for the random intercept of'subject', suggesting that individual differences among subjects play a substantial role in the observed categorization rates. Concerning the fixed effects, abstract-philosophical, concrete-artifact, concrete-natural and interoceptive ratings exhibit statistically significant effects on classification rate (*p* < 0.05). Notably, the measure of interoceptive ratings shows a significant positive association with classification rate (Estimate = 0.013706, *p* = 0.015162). The detailed output is reported in the Supplementary Materials.

The ANOVA generated from the GLMMs model indicates that Stimulus Category accounts for the largest proportion of variance in the categorization rates variable, suggesting it is the most influential predictor in the model (*F*-value = 19.293, SumSq = 57.880, MeanSq = 19.293). IAc also plays a significant contribution (*F*-value = 8.479, SumSq = 8.479) as interoceptive ratings (*F*-value = 5.768, SumSq = 5.768). The interaction term between Stimulus Category and IAc also contributes significantly (*F*-value = 6.557, SumSq = 19.670) indicating that the effect of IAc on the categorization rates varies depending on the Stimulus Category.

The Emmeans package for testing interaction pairs enables the determination of significant differences in categorization rates across stimulus category levels, accounting for the influence of the two covariates mean IAc scores and interoceptive rating values. The analysis (p-adjusted with the Boferroni method) indicates a lower likelihood of misclassifying abstract-emotional concepts than abstract-philosophical concepts (Estimate = −0.878, SE = 0.163, z.ratio _Emo_Phil_ = −5.380, *p* < 0.001), and concrete-artifact than concrete-natural concepts (Estimate = −0.882, SE = 0.179, z.ratio _Art_Nat_ = −4.93, *p* < 0.001). The between-category comparison indicates lower probability of alternative classifications for abstract-emotional concepts compared to concrete-natural (Estimate = −0.733, SE = 0,278, z.ratio _Emo_Nat_ = −2.641, *p* = 0.049), but no difference between abstract-emotional vs concrete-artifact concepts (Estimate = 0.148, SE = 0.307, z.ratio _Emo_Art_ = 0.484, *p* = 1). Abstract-philosophical concepts had a greater probability of alternative classification than concrete-artifact concepts (Estimate = 1.027, SE = 0.234, z.ratio _Phil_Art_ = 4.381, *p* = 0.001), but not concrete-natural concepts (Estimate = 0.145, SE = 0.200, z.ratio _Phil_Nat_ = 0.725, *p* = 1).

In summary, these results indicate that abstract-emotional concepts are categorized with greater consistency than abstract-philosophical concepts, and concrete-artifact concepts demonstrate a higher level of categorization consistency than concrete-natural concepts. The significant interaction between stimulus category and IAc suggests that the influence of interoception on categorization varies across different stimulus types. Specifically, when interoception is explicitly considered, as in the Emmeans analysis, we observe distinct patterns of categorization rates across stimulus categories. In addition, the between-category comparisons (which do not explicitly model IAc and interoceptive ratings), also show significant differences, suggesting that while interoception plays a role, there are also category-specific effects that exist independently. Thus, both the intrinsic properties of the stimulus categories and individual differences in interoceptive abilities appear to influence categorization performance.

## Discussion

This study presents several key points that provide methodological and theoretical insights to better understand the interplay between interoceptive processes and conceptual representations. The first key point is methodological, as we have developed and tested a new behavioral paradigm that allows us to elucidate the implicit activation of interoceptive features in the categorization of concepts. The interoceptive-exteroceptive categorization paradigm that we have developed creates a door to the representation of concepts, highlighting the malleability of the boundaries between different types of concepts and the multiplicity of dimensions involved in their conceptual knowledge (see also Fiorillo & Gorwood, 2020; Moccia et al., 2020). Notably, participants performed the task with a high degree of accuracy despite the ambiguity implicit in the request to categorize concepts on the interoceptive-exteroceptive axis, creating a substantially artificial dichotomy.

The second key point is theoretical and concerns the contribution of the study results to embodied theories and the debate on the representation of abstract concepts. Importantly, they show that concepts are embodied not only to the extent that they evoke sensorimotor experiences but also because they evoke and enhance inner, interoceptive experiences. For the first time, this is shown with an implicit, categorization task. This new task was complemented by individuals’ measures of interoceptive accuracy rather than relying solely on self-reported measures.

Overall, the participants in this study had poor cardiac interoceptive accuracy. This is not new to the field as the individual ability to detect interoceptive signals largely varies and may be influenced by stress and adverse life experiences that negatively affect the attending and appraisal of inner bodily sensations (see, for example, Calì et al., 2015).

The MAIA results reveal a pattern of heightened self-reported interoceptive sensibility (high Noticing) alongside lower self-reported interoceptive regulation. Participants indicated a strong awareness of their bodily sensations but reported a lesser tendency to manage emotional responses based on these sensations. This pattern of subjective beliefs and tendencies may suggest a potential vulnerability to increased anxiety and emotional distress, highlighting the importance of not just noticing bodily signals, but also the perceived difficulty in regulating the emotional responses they evoke. This reported difficulty in regulating emotional responses to bodily sensations aligns with the participants’ affective profile, which indicated elevated levels of alexithymia (particularly a tendency toward externally oriented thinking and difficulty in recognizing emotions), with medium to high levels of anxiety and depression. Although these data are unexpected, it is reasonable to think that they may be, at least, partly due to the particular conditions we have experienced in recent years. The high values of anxiety and negative affectivity in the population might be an after-effect of the traumatic event that we have collectively experienced due to the recent pandemic. Indeed, numerous studies have documented the ‘affective sequelae’ of the COVID-19 outbreak, during the lockdown (Fiorillo & Gorwood, 2020) and up to one year after the emergence (Barca et al., 2025). Furthermore, the current political and economic situation generated by the Russian-Ukrainian conflict might be an additional contextual stressor (Barchielli et al., 2022) that has been shown to affect the psychological well-being of young adults (Regnoli et al., 2024).

Notably, this study is complemented also with interoceptive ratings as a measure of the conceptual representation of interoception. These ratings also capture the relationship between interoceptive features and other key conceptual dimensions, including abstractness, concreteness, body-object interaction and emotionality, demonstrating that interoceptive ratings not only measure the interoceptive grounding of concepts but also provide a comprehensive view of how these concepts are situated within a broader semantic space. Overall, we observe a complex interplay between these variables: interoceptive ratings appear to be positively linked to both abstractness and emotionality, with more abstract and emotional stimuli eliciting higher interoceptive ratings. However, they are negatively related to concreteness and BOI. Crucially, all variables are significantly correlated with each other (see Table 2). This is not entirely unexpected, as these constructs are likely to be related, as also appears in other Italian databases (e.g., Villani et al., 2019). For example, it is reasonable to assume that abstractness and concreteness, being opposite ends of a spectrum, would exhibit a strong negative correlation. Similarly, emotionality and interoceptive ratings might be linked, as emotional experiences often involve heightened awareness of bodily states. However, while these correlations provide valuable insights into the relationships between the constructs, they also suggest that there might be shared underlying factors influencing these ratings. For instance, a general tendency to focus on internal sensations could potentially influence both interoceptive and emotionality ratings.

While we have explored bivariate correlations here, future analyses with a greater sample of stimuli and employing multivariate techniques should carefully consider and address potential multicollinearity issues to disentangle the specific roles of each construct. Despite these considerations, the observed correlations offer valuable preliminary evidence regarding the interplay between abstractness, concreteness, body-object interaction, emotionality, and interoceptive ratings.

Concerning conceptual representations, the pattern of results of the novel Interoceptive-Exteroceptive Judgement task described here is rather interesting and straightforward.

A key finding is the high degree of convergence among participants in categorizing concrete-artifact concepts as exteroceptive. Classifying them as ‘exteroceptive ‘requires less time than all the other categories and subcategories considered here. From a kinematic point of view, their mouse trajectories have the smallest paths toward the exteroceptive response, the smaller Area Under the Curve, and Maximum Deviation, indicating reduced uncertainty in categorizing them compared with concrete-natural concepts and abstract-philosophical and abstract-emotional concepts. In addition, concrete-artifact concepts are less affected than concrete-natural concepts by interoceptive accuracy. Differently, concrete-natural concepts were more often subject to alternative interoceptive classifications than concrete-artifact concepts, indicating reduced congruency in how these categories are conceptually represented. This result indicates that concrete concepts cannot be represented as unitary if we consider the exteroceptive/interoceptive dimensions they evoke. Interestingly, natural kinds seem to evoke interoceptive features more than artifacts do, similar to abstract concepts. Notably, the concrete-natural concepts we selected do not refer to animals or food but rather to natural scenes and elements (e.g., cave, forest, ocean) that might evoke emotions such as awe, fear, and wonder. Interestingly, our results offer insights into studies examining the beneficial effects of nature and natural stimuli on cognitive activity. For example, recent studies have shown that people with high sensory processing sensitivity (SPS) to outer and inner stimuli tend to feel more connected to nature, experience awe more frequently, and more frequently adopt pro-environmental behaviors (Dunne et al., 2024) In the present study, natural contexts and scenes did not elicit consistent exteroceptive categorizations from participants, suggesting a complex interplay of interoceptive and exteroceptive features.

Notably, regarding abstract concepts, participants showed high convergence in categorizing abstract-emotional concepts as interoceptive. As predicted, abstract-emotional concepts evoked stronger interoceptive features, resulting in a lower likelihood of exteroceptive classification compared to philosophical concepts. This finding is interesting for several reasons. First, it confirms the relevance of the interoceptive dimension across various abstract concept types, even though its influence extends to certain concrete concepts as well. However, it also highlights the particular criticality of the interoceptive dimension for emotional concepts, a specific type of abstract concept (Winter, 2022).

Overall, our findings, which incorporate interoceptive and exteroceptive dimensions in conceptual representations, suggest that the distinction between concrete and abstract concepts is not a simple dichotomy. Rather, concrete-natural objects evoking emotions display a closer resemblance to abstract concepts than to other concrete concepts, even though their referents are concrete entities, unlike those of abstract concepts.

## Limitations of the study

One limitation of the present study is the exclusive recruitment of female participants, a decision made to control for potential confounding variables given our sample size. Research highlights biological sex-related differences in interoceptive sensibility (Barca et al., 2025; Grabauskaitė et al., 2017; Naraindas et al., 2024). For instance, Naraindas and colleagues (Naraindas et al., 2024) found that women reported significantly higher scores on measures of attention regulation and reduced worry towards visceral signals, while men exhibited stronger capabilities in attention regulation, body listening, and body trusting. Regarding interoceptive accuracy, a meta-analysis by (Prentice & Murphy, 2022) revealed clear sex differences in cardiac and respiratory interoception, but not in gastric interoception, with males performing better than females. However, a recent study by (Haruki et al., 2025), employing a modified heartbeat counting task with numerous trials and controlling for heart rate and trial duration, found no significant gender differences in interoceptive accuracy. Instead, they highlighted under confidence in women's performance, leading to lower metacognitive awareness of their accuracy, suggesting that previous reports of lower accuracy in women might be confounded by differing heart rates and this tendency for underreporting confidence. This nuanced view suggests that while the fundamental ability to perceive internal signals may not differ, confidence and evaluation of these perceptions could vary by gender. These findings underscore the complex and domain-specific nature of biological sex influences on interoception. To mitigate the potential confounding effect of these baseline interoceptive variations between male and females, which could have obscured the effects of interest, the present study focused solely on female participants. We acknowledge that this methodological choice may limit the generalizability of our findings. Future research should prioritize the inclusion of both sexes to provide a more comprehensive understanding of these relationships.

Furthermore, while we employed the widely used Heartbeat Counting Task (HBCT) to assess cardiac interoceptive accuracy, we acknowledge as a limitation the absence of an additional measure, such as the Heartbeat Discrimination Task, which could have served as a control to further strengthen the robustness of our findings.

Also, the participants enrolled in the study were visitors to the National Library of Rome. Thus, they might represent the general population to a greater extent than the typical study population enrolling university students. This might account for higher scores on the depression questionnaire and the reduced interoceptive accuracy than those reported in studies employing psychology students.

Another final concern is the completion of the questionnaires at home. This choice, motivated by the need to reduce the duration of the experimental session, however, meant that some participants did not complete all the questionnaires, probably due to a decrease in motivation.

## Conclusions

Overall, our findings indicate that the semantic knowledge associated with interoceptive experiences contributes to the conceptual representation of a wide range of concepts. Importantly, while it is crucial for abstract concepts, it also plays a major role in the categorization of concrete and natural concepts, at least for those representing natural scenery. Hence, the study questions the rigid separation between concrete and abstract concepts in terms of interoception and exteroception and highlights the fundamental flexibility and task dependence of human conceptual representation.

## Supplementary Information

Below is the link to the electronic supplementary material.

Supplementary file1 (DOCX 29 KB)

Supplementary file2 (DOCX 32 KB)

## Data Availability

The datasets used and analyzed during the current study are available from the corresponding author on reasonable request.
