# Supplementary material for: Interoceptive grounding of conceptual knowledge: new insight from an interoceptive-exteroceptive categorization task of concepts
Source: Psychol Res. 2026 Jan 3;90(1):13. doi: 10.1007/s00426-025-02155-8 (PMC12764517; doi:10.1007/s00426-025-02155-8)
Supplement: Supplementary file 1 — Supplementary file1 (DOCX 29 KB) [file 426_2025_2155_MOESM1_ESM.docx]

**Appendix**

| **STIMULUS**  **(Italian)** | **STIMULUS**  **(English translation)** | **STIM-CAT** | **ABS** | **CONC** | **INTERO** | **EMO** | **BOI** |
| --- | --- | --- | --- | --- | --- | --- | --- |
| **AEREO** | **AIRPLANE** | con-art | 9.22 | 86.03 | 18.03 | 24.03 | 36.88 |
| **ARGENTO** | **SILVER** | con-nat | 12.08 | 83.11 | 7.88 | 10.53 | 80.84 |
| **AUTOMOBILE** | **CAR** | con-art | 6.50 | 85.09 | 20.18 | 13.97 | 74.00 |
| **BELLEZZA** | **BEAUTY** | abs-emo | 52.31 | 41.86 | 55.91 | 55.50 | 42.13 |
| **CALMA** | **CALM** | abs-emo | 65.44 | 44.00 | 64.61 | 61.47 | 40.63 |
| **CAVERNA** | **CAVE** | con-nat | 16.50 | 79.83 | 9.36 | 23.91 | 64.06 |
| **CHIESA** | **CHURCH** | con-art | 27.86 | 68.23 | 8.55 | 22.44 | 80.34 |
| **CONFLITTO** | **CONFLICT** | abs-emo | 51.67 | 48.97 | 64.88 | 55.59 | 48.53 |
| **CRISTALLO** | **CRISTAL** | con-nat | 11.86 | 83.31 | 11.94 | 10.18 | 67.59 |
| **DESTINO** | **DESTINY** | abs-phr | 80.56 | 20.63 | 55.48 | 61.29 | 28.94 |
| **DIAMANTE** | **DIAMOND** | con-nat | 8.22 | 83.94 | 10.42 | 13.50 | 68.13 |
| **ELICOTTERO** | **HELICOPTER** | con-art | 6.17 | 86.57 | 19.21 | 9.00 | 59.97 |
| **ENIGMA** | **ENIGMA** | abs-phr | 61.78 | 39.00 | 32.79 | 34.94 | 28.19 |
| **FORBICI** | **SCISSORS** | con-art | 9.39 | 85.74 | 10.39 | 9.03 | 62.88 |
| **FORCHETTA** | **FORK** | con-art | 6.72 | 86.86 | 11.27 | 5.44 | 80.41 |
| **FOSCHIA** | **MIST** | con-nat | 21.44 | 67.26 | 19.79 | 21.76 | 55.00 |
| **GIOIA** | **JOY** | abs-emo | 59.08 | 36.69 | 64.18 | 71.06 | 46.97 |
| **GIUDIZIO** | **JUDGEMENT** | abs-phr | 56.00 | 37.37 | 58.42 | 70.97 | 40.28 |
| **INFANZIA** | **CHILDHOOD** | abs-emo | 40.08 | 55.09 | 61.45 | 70.85 | 37.53 |
| **LOGICA** | **LOGIC** | abs-phr | 63.72 | 47.63 | 34.09 | 30.44 | 28.72 |
| **MARTELLO** | **HAMMER** | con-art | 8.25 | 82.94 | 7.79 | 10.06 | 79.69 |
| **MINERALE** | **MINERAL** | con-nat | 9.81 | 77.14 | 7.09 | 8.09 | 65.50 |
| **MORALE** | **MORAL** | abs-phr | 74.36 | 33.17 | 16.30 | 54.21 | 71.75 |
| **MOTIVO** | **REASON** | abs-phr | 60.11 | 41.46 | 49.18 | 43.24 | 25.66 |
| **NEGOZIO** | **SHOP** | con-art | 7.47 | 84.09 | 9.85 | 12.82 | 71.38 |
| **OCEANO** | **OCEAN** | con-nat | 19.94 | 73.00 | 39.24 | 53.21 | 74.28 |
| **PALMA** | **PALM** | con-nat | 9.69 | 85.57 | 13.39 | 12.82 | 65.69 |
| **PALUDE** | **SWAMP** | con-nat | 10.17 | 76.80 | 14.06 | 18.06 | 62.75 |
| **PARADISO** | **PARADISE** | abs-phr | 83.78 | 20.89 | 37.42 | 41.62 | 26.34 |
| **PECCATO** | **SIN** | abs-phr | 66.75 | 32.29 | 38.06 | 42.85 | 13.19 |
| **PENNELLO** | **BRUSH** | con-art | 8.92 | 85.71 | 10.33 | 19.53 | 76.72 |
| **PINETA** | **PINE** | con-nat | 8.36 | 78.71 | 16.52 | 27.03 | 66.84 |
| **PUNIZIONE** | **PUNISHMENT** | abs-emo | 42.22 | 48.20 | 47.15 | 48.76 | 48.84 |
| **SALVEZZA** | **SALVATION** | abs-phr | 66.33 | 36.20 | 40.12 | 54.24 | 35.69 |
| **SCHEMA** | **SCHEME** | abs-phr | 35.83 | 58.37 | 47.42 | 22.38 | 31.81 |
| **SCOPA** | **BROOM** | con-art | 9.19 | 85.60 | 14.94 | 8.21 | 77.34 |
| **SCOPERTA** | **DISCOVERY** | abs-emo | 50.58 | 42.29 | 48.97 | 52.41 | 35.41 |
| **SFORZO** | **EFFORT** | abs-emo | 39.03 | 60.80 | 52.36 | 46.03 | 65.38 |
| **SICUREZZA** | **SECURITY** | abs-emo | 54.81 | 39.57 | 59.76 | 63.03 | 43.75 |
| **VENDETTA** | **REVENGE** | abs-emo | 55.78 | 44.69 | 52.45 | 48.47 | 40.47 |

**Legend:** STIM-CAT = Stimulus-category; Con-art= Concrete-artifact; Con-nat=Concrete-natural; Abs-emo= Abstract-emotional; Abs-phr= Abstract philosophical; ABS= Abstractness; CONC= Concreteness; INTERO= Interoceptive ratings; EMO=Emotionality; BOI= Body Object Interaction.
