# Supplementary material for: Interoceptive grounding of conceptual knowledge: new insight from an interoceptive-exteroceptive categorization task of concepts
Source: Psychol Res. 2026 Jan 3;90(1):13. doi: 10.1007/s00426-025-02155-8 (PMC12764517; doi:10.1007/s00426-025-02155-8)
Supplement: Supplementary file 2 — Supplementary file2 (DOCX 32 KB) [file 426_2025_2155_MOESM2_ESM.docx]

**Supplementary Materials**

**1.1 Linear Mixed-Effects Model Selection for Reaction Time Data**

The first model (RT ~ Stimulus Category * Response Position * IAc + (1+IAc|subject) + (1 | trial)) is designed to investigate how the combined effects of Stimulus Category, Response Position, and Interoceptive Accuracy (IAc) influence response time, while taking into account the fact that RTs are likely to vary both between subjects (some are faster than others) and between trials (some trials might be easier or more attention-grabbing than others). The inclusion of a random slope for IAc allows the model to capture the possibility that the influence of interoceptive accuracy itself might differ across individuals. Since this model produced a "boundary (singular) fit" warning, we simplified it by 1) removing the interaction effects involving Response Position, and 2) simplifying the random effects structure, removing the random slope for IAc. Next, we compared the first simplified model (with the Response Position interaction) to the second simplified model (with the simpler random effects) using an ANOVA. A p-value of 0.044 suggests that there is a statistically significant difference between the two models. Since the simpler model (which includes the interaction between Stimulus Category and IAc, random intercepts for subjects and trials) have a *lower* BIC (32237 vs 32283), and the ANOVA suggests a significant difference, is the final model we adopted.

The final model adopted includes the interaction between Stimulus Category and Interoceptive Accuracy (IAc), with random intercepts for subjects and trials. Specifically, it can be represented as: RT ~ Stimulus Category * IAc + (1 | subject) + (1 | trial).

| **RT** | | | | **Init Time** | | | **AUC** | | | **MD** | | |
| --- | --- | --- | --- | --- | --- | --- | --- | --- | --- | --- | --- | --- |
| *Predictors* | *Estimates* | *CI* | *p* | *Estimates* | *CI* | *p* | *Estimates* | *CI* | *p* | *Estimates* | *CI* | *p* |
| (Intercept) | 1732.32 | 1573.23 – 1891.40 | <0.001 | 454.45 | 329.16 – 579.73 | <0.001 | 0.12 | -0.04 – 0.29 | 0.147 | 0.21 | 0.06 – 0.37 | 0.007 |
| abs-phr | 19.07 | -46.44 – 84.58 | 0.568 | 31.23 | -40.29 – 102.75 | 0.392 | -0.10 | -0.34 – 0.15 | 0.448 | -0.01 | -0.24 – 0.22 | 0.954 |
| con-art | -95.56 | -156.94 – -34.18 | 0.002 | 23.40 | -44.60 – 91.41 | 0.500 | -0.34 | -0.57 – -0.10 | 0.005 | -0.49 | -0.71 – -0.27 | <0.001 |
| con-nat | 71.24 | 6.76 – 135.72 | 0.030 | 5.15 | -65.25 – 75.55 | 0.886 | -0.04 | -0.28 – 0.21 | 0.756 | -0.32 | -0.54 – -0.09 | 0.006 |
| IAc | -125.63 | -453.19 – 201.93 | 0.452 | -162.04 | -386.90 – 62.83 | 0.158 | -0.15 | -0.49 – 0.18 | 0.367 | -0.37 | -0.70 – -0.05 | 0.025 |
| abs-phr * IAc | -14.77 | -145.78 – 116.24 | 0.825 | -62.90 | -214.36 – 88.56 | 0.415 | 0.22 | -0.27 – 0.72 | 0.382 | -0.11 | -0.59 – 0.37 | 0.655 |
| con-art * IAc | 76.97 | -45.88 – 199.81 | 0.219 | -20.49 | -163.50 – 122.52 | 0.779 | 0.35 | -0.12 – 0.82 | 0.142 | 0.73 | 0.27 – 1.19 | 0.002 |
| con-nat * IAc | -106.08 | -234.31 – 22.15 | 0.105 | -33.96 | -185.46 – 117.55 | 0.660 | 0.04 | -0.45 – 0.53 | 0.873 | 0.72 | 0.25 – 1.19 | 0.003 |
| **Random Effects** |  | | | | | | | | | | | |
| σ^2^ | 63256.37 |  |  | 91883.34 |  |  | 0.98 |  |  | 0.96 |  |  |
| τ _00 trial_ | 8888.25 |  |  | 3368.28 |  |  | 0.00 |  |  | 0.01 |  |  |
| τ _00 subject_ | 52361.80 |  |  | 53572.82 |  |  | 0.00 |  |  | 0.00 |  |  |
| ICC | 0.49 |  |  |  |  |  | 0.00 |  |  |  |  |  |
| τ _11 subject.HBT_ |  |  |  | 1450.69 |  |  |  |  |  | 0.00 |  |  |
| ρ _01 subject_ |  |  |  | -1.00 |  |  |  |  |  |  |  |  |
| N _subject_ | 38 |  |  | 42 |  |  | 38 |  |  | 42 |  |  |
| N _trial_ | 80 |  |  | 80 |  |  | 80 |  |  | 80 |  |  |
| Observations | 2295 |  |  | 2212 |  |  | 2326 |  |  | 2212 |  |  |
| Marginal R^2^/ Conditional R^2^ | 0.018 / 0.501 |  |  | 0.024 / NA |  |  | 0.007 / 0.010 |  |  | 0.015 / NA |  |  |

**1.2 LMMs Output: Detailed Statistical Results. Abs-phr: abstract philosophical, con-art: concrete natural, con-nat: concrete natural, IAc: interoceptive accuracy; INTERO: interoceptive ratings.**

**1.3 General Linear Mixed-Effects model selection for categorization rates**

Categorization rates were analyzed using a generalized linear mixed-effects model (GLMM) with a binomial distribution, modeling the binary response data (0 = dominant response, 1 = alternative response). The initial model included the interaction between Stimulus Category and IAc, the fixed effect of interoceptive ratings, and random intercepts for subject and trial. Since the model encountered convergence issues, we first refitted it using the 'bobyqa' optimizer with an increased maximum function evaluation limit. This refitted model resulted in a boundary (singular) fit, suggesting potential issues with the random effects structure.

Thus, we created a simpler model excluding the random intercept for 'trial'. Model comparison (ANOVA) indicated no significant difference between the two models (Chi-square = 0, p-value = 1) suggesting that the inclusion of the random effect for 'trial' did not significantly improve the model fit. Thus, the final model included fixed effects for Stimulus Category (categorical), IAc (continuous), interoceptive ratings (continuous), and their interaction, along with a random intercept for subject. The model was fitted using maximum likelihood with Laplace approximation and the 'bobyqa' optimizer. The summary output of the model (see section 1.2) provides a comprehensive overview of the generalized linear mixed model's performance.

The model demonstrates a reasonable fit, as evidenced by the AIC and BIC values, which balance model fit with complexity. The random effects section highlights significant inter-subject variability, with a variance of 0.603 for the random intercept of 'subject', suggesting that individual differences among subjects play a substantial role in the observed categorization rates. Concerning the fixed effects, abstract philosophical, concrete artifact and natural concepts, and also interoceptive ratings exhibit statistically significant effects on categorization rates (p < 0.05). Notably, interoceptive ratings shows a significant positive association with categorization rates (Estimate = 0.013706, p = 0.015162), suggesting that higher interoceptive ratings are associated with increased alternative categorization rates. Furthermore, significant interaction effects between concrete artifact and IAc, and concrete natural concepts and IAc were observed.

**1.2 GLMs Output on categorization rates. Abs-phr: abstract philosophical, con-art: concrete natural, con-nat: concrete natural; IAc: interoceptive accuracy; INTERO: interoceptive ratings.**

|  |  | **Categorization rates** |  |
| --- | --- | --- | --- |
| *Predictors* | *Odds Ratios* | *CI* | *p* |
| (Intercept) | 0.12 | 0.05 – 0.29 | <0.001 |
| abs-phr | 2.89 | 1.61 – 5.17 | <0.001 |
| con-art | 3.27 | 1.48 – 7.21 | 0.003 |
| con-nat | 4.54 | 2.17 – 9.49 | <0.001 |
| IAc | 0.46 | 0.11 – 1.90 | 0.286 |
| INTERO | 1.01 | 1.00 – 1.03 | 0.015 |
| abs-phr * IAc | 0.65 | 0.20 – 2.16 | 0.485 |
| con-art * IAc | 0.04 | 0.01 – 0.21 | <0.001 |
| con-nat * IAc | 0.16 | 0.04 – 0.59 | 0.006 |
|  |  |  |  |
| **Random Effects** |  |  |  |
| σ2 | 3.29 |  |  |
| τ00 subject | 0.60 |  |  |
| ICC | 0.15 |  |  |
| N subject | 38 |  |  |
| Observations | 2901 |  |  |
| Marginal R2 / Conditional R2 | 0.131 / 0.265 |  |  |
